# Multi-perspective hotel operation process anomaly prediction method based on graph transformer and autoencoder

**DOI:** 10.3389/frai.2025.1682701

**Published:** 2025-12-16

**Authors:** Yidan Ma, Yue Wu, Xinsheng Fang

**Affiliations:** 1 School of Economics and Trade Management, Anhui Vocational College of Defense Technology, Luan, China; 2 School of Economics and Management, Tiangong University, Tianjin, China; 3 School of Mathematics and Big Data, Anhui University of Science and Technology, Huainan, China

**Keywords:** hotel operation, process anomaly prediction, graph transformer, behavioral relationship, behavioral footprint

## Abstract

Due to the complexity of hotel operation processes, abnormal situations are inevitable, making proactive anomaly prediction essential for ensuring operational stability. Although current deep learning methods can encode control and data flows to predict anomalies in attributes like activity and time, they often fail to adequately represent the behavioral relationships between activities and lack specific mechanisms to model the interaction between control and data flows. To address these challenges, this paper proposes a business process anomaly prediction method based on a Multi-perspective Graph Transformer and Auto Encoder (MLGTAE). The proposed method first constructs multi-perspective trace graphs by combining Petri nets—which capture process behaviors—with data attributes such as time and resources. It then leverages an attention mechanism to achieve deep semantic interaction between process behavior and data, followed by a decoder that performs reconstruction to detect anomalies. Validated on multiple real-world datasets, the results demonstrate that MLGTAE outperforms existing state-of-the-art methods, showing superior accuracy in predicting anomalies at both the activity and data attribute levels.

## 1 Introduction

In the backdrop of the intensifying competition within the contemporary hotel industry, hotel groups are compelled to guarantee the seamless operation of their business processes to elevate customer satisfaction and operational efficiency. Nevertheless, during the daily operations of hotels, the emergence of anomalies is inevitable. Examples of such anomalies include duplicate reservations, fraudulent reservations, and unprocessed cancellations. These irregularities not only impede the operational efficiency of hotels but also have the potential to cause a decline in customer satisfaction. To mitigate the losses incurred due to process anomalies, the most prudent strategy is to identify latent anomalies in advance. By leveraging anomaly prediction techniques to detect the likelihood of anomalies beforehand and implementing corresponding anomaly management measures, the normal functioning of hotel business processes can be safeguarded. This not only facilitates the enhancement of hotel groups' management efficiency and reduction of manual processing costs but also offers customers a superior service experience.

Anomaly prediction, a crucial subfield within business process prediction and monitoring, primarily aims to detect potential deviations, omissions, and the like. These irregularities can be induced by activity execution delays and external factors amidst a vast number of normal process activities (Goldstein and Uchida, 2016). With the ongoing evolution of information systems, the technology of anomaly prediction has garnered escalating attention. Given the well-established scientific principle that low-quality event logs yield subpar results (Ko and Comuzzi, 2023), anomaly prediction has been extensively developed and applied across diverse domains, including smart healthcare, industrial manufacturing, and financial fraud detection.

Early investigations into business process anomaly prediction predominantly centered on the control flow level. These studies emphasized the structural details of business process models and utilized standard business process reference models to forecast anomalies during activity execution via replay or alignment (Tavares and Barbon, 2023). With the advancement of machine learning and deep learning, the integration of learning techniques into the domain of control flow anomaly prediction has significantly enhanced the prediction efficiency. Notably, beyond activity information, event logs encompass a substantial volume of data attribute information, including the execution time and resources associated with activities. Focusing solely on control flow level anomalies may overlook scenarios where activities with reasonable execution are accompanied by unreasonable attribute values. To enable multi-perspective anomaly prediction of business processes, recent research has employed encoding techniques to represent both control flow and data flow information within event logs. Subsequently, deep learning models are trained using multi-dimensional information (Huo et al., 2021) to conduct multi-perspective anomaly prediction.

While current multi-perspective models enable rapid prediction, they often neglect fine-grained behavioral relationships among activities and provide insufficient consideration of the interaction between data flow and control flow. Graph deep learning (Guan et al., 2023a,b) has emerged as a prominent research domain in recent times. Representing the behavioral relationships of business processes through graph encoding is a viable approach. However, basic graph deep learning models exhibit limitations in handling graph-represented long sequences and global information attention. Notably, the attention mechanism of the Transformer model can effectively address these challenges. Consequently, a substantial number of Graph Transformer (Cai and Lam, 2020; Yun et al., 2019; Nguyen et al., 2022) models have been proposed. These models integrate Transformer and graph deep learning models to achieve complementary advantages and find applications in diverse fields. Although methods such as GAE (Huo et al., 2021) employ graph autoencoders, they typically construct graphs based on simple “direct-follow” relationships, thereby losing complex semantic behaviors. Models like BINet (Nolle et al., 2022) and GRASPED (Guan et al., 2023a,b), although integrating multi-dimensional data, mainly adopt RNN/GRU architectures, making it difficult to capture long-range dependencies and complex graph structures. Our MLGTAE model uniquely addresses these issues by constructing semantically rich graphs using behavioral footprints extracted from Petri nets, rather than just direct-follow relationships; and by using a graph transformer as the encoder, which combines the advantages of GCN (for handling graph structures) and Transformer (for capturing global information).

In response to the existing problems in anomaly prediction research, this paper proposes a multi-perspective business process anomaly prediction method based on Graph Transformer and autoencoder. The main contributions of this method are:

1) We utilize Petri nets to extract the process's behavioral footprint. By constructing a behavioral relationship matrix and integrating data attributes from event logs, we achieve a unified multi-perspective information representation containing fine-grained behavioral relationships.
2) A novel anomaly prediction method based on Graph Transformer and autoencoder is proposed. It integrates a Graph Transformer (combining GCN and Transformer) capable of handling complex graph structures while capturing global dependencies, thereby achieving effective multi-perspective anomaly prediction.
3) Extensive experiments are conducted using real-world data and compared with multiple advanced methods to verify the impact of fine-grained behavioral information in business processes on anomaly prediction tasks, and to demonstrate the anomaly prediction performance of the proposed method at both data and behavioral levels.

The remaining structure of this article is as follows: Section 2 mainly introduces the related work in the field of business process anomaly detection; Section 3 mainly introduces some basic concepts that are needed to propose the method in this paper; Section 4 mainly elaborates on the method proposed in this paper in detail; Section 5 evaluates the proposed method using real datasets to verify its feasibility; Section 6 mainly summarizes the work of this paper and describes possible future research directions.

## 2 Related work

### 2.1 Single-view prediction technology

Regarding the research on anomaly prediction that solely concentrates on a single level of control flow or data attributes, Bezerra and Wainer (2013) put forward four anomaly prediction algorithms. Their work placed a specific emphasis on trace-level anomalies, including infrequent traces and traces that deviated from the regular path within logs. They validated the effectiveness of their methods by utilizing artificially synthesized event logs. Rudolf et al. (2024) tackled the anomaly prediction problem in multi-case interaction. They proposed an anomaly prediction approach that integrated classification networks and autoencoders. This method centered on the interaction information among cases and the context of the current node, thereby enabling anomaly prediction across case interactions. Saini et al. (2020) presented an anomaly prediction framework grounded in process mining technology. This framework employs the proposed process discovery and consistency checking algorithms to detect and interpret anomalies within the control flow of business processes. Van der Aa et al. (2021) exploited natural language processing techniques to forecast behavioral anomalies in business process event logs. The proposed approach centers on the behavioral semantics among activities, extracting business objects and operations from activity labels and identifying abnormal patterns through comparison with domain-relevant knowledge bases. Nolle et al. (2018) put forward a business process anomaly prediction method grounded in autoencoders, thereby demonstrating the advantages of autoencoders in anomaly prediction. This method is capable of predicting anomaly information at the event level without the necessity of prior knowledge for model training. Junior et al. (2020) tackled the challenge of the inability to acquire anomaly labels in real-world environments for model training and anomaly prediction by proposing an anomaly prediction method that integrates natural language encoding technology and classification algorithms. This method maps activities to vector spaces by means of natural language processing techniques and subsequently accomplishes anomaly prediction via classification algorithms. Furthermore, Lawal et al. (2024) have focused on developing intelligent anomaly detection methods, particularly addressing challenges like imbalanced data in process logs.

### 2.2 Multi-perspective prediction technology

Research on multi-perspective anomaly prediction involving control flow and data attributes, Sarno et al. (2020) utilized a fuzzy multi-attribute decision-making approach to determine the anomaly probability of business process execution, and combined fuzzy association rules and process mining techniques to learn interaction rules in process logs, thereby achieving rapid anomaly prediction. This method not only focuses on the control flow dimension but also on the data information of activity execution. However, this approach requires a standard business process to analyze event logs. Boldt et al. (2020) proposed a Markov chain-based event sequence anomaly prediction method, which starts from the time dimension and focuses on abnormal changes across multiple time spans. Pauwels and Calders (2019) to address the issue of handling unseen activities or attributes in business processes, proposed an optimization method of dynamic Bayesian networks, which can achieve anomaly scoring and explanation for new values. Nguyen et al. (2019) utilized autoencoders to predict and repair anomalies at the attribute level, and verified the usability of the proposed method through synthetic and real data, and explored the impact of anomalies and missing values on the variability of event logs. Nolle et al. (2022) proposed an anomaly prediction method—Binet, which integrates the control flow perspective and data flow perspective. This method is based on recurrent neural networks to achieve dynamic and multi-perspective business process anomaly prediction, and designed a heuristic anomaly threshold setting method based on human intuition, solving the limitation of manual threshold setting in traditional anomaly prediction. Ko and Comuzzi (2021) utilized statistical leverage techniques from information theory to predict anomalies in business processes. Different from traditional information theory distance measurement methods, this method considers the problem of different feature magnitudes in different process traces, improving its performance at the attribute level. Elaziz et al. (2023) applied deep reinforcement learning to the field of business process anomaly prediction, using a small amount of anomaly data in a weakly supervised manner to improve anomaly prediction accuracy, and combined the reward mechanism of reinforcement learning with variational autoencoders to achieve identification and prediction of unknown anomaly types. Guan et al. (2023a),b proposed an anomaly prediction architecture that integrates bidirectional gated recurrent units and autoencoders, which uses attention mechanisms and teacher forcing methods to enhance anomaly prediction for business process control flow and data flow. Experiments show that the proposed method outperforms other methods in multiple perspectives. Böhmer and Rinderle-Ma (2020) addressed several issues in business process anomaly prediction, such as false alarms, flexible detection, and root cause analysis, and proposed an anomaly prediction method based on association rules, which uses dynamic anomaly thresholds and lenient strategies to achieve dynamic calculation of rule influence. Krajsic and Franczyk (2021) proposed an anomaly prediction method that combines attention mechanisms and classification models, based on an autoencoder architecture and bidirectional long short-term memory networks to achieve semi-supervised anomaly prediction. This method shows good performance in time series and business process event-level task processing.

### 2.3 Detection technology for behavioral attention

In the realm of business process management, process models constitute an indispensable and pivotal component. Their significance resides in the capacity to formalize the behavioral relationships among activities, a task that proves arduous for sequence-based deep learning methods [such as Recurrent Neural Networks (RNN) and Long Short-Term Memory networks (LSTM)] to capture directly. Consequently, a body of research has started to center on the behavioral information within business processes. Lu et al. (2016) tackled the issue of activity-level anomaly prediction in the absence of a pre-defined process reference model by detecting anomalies via behavioral similarity mapping. Genga et al. (2018) concentrated on the anomaly problems that might emanate from the parallel behavioral relationships among business process activities and put forward a correlation subgraph-based anomaly prediction approach. This approach constructs correlation subgraphs encompassing parallel relationships using partially ordered logs and identifies frequently occurring anomalies in these subgraphs through consistency detection methods, which are subsequently formalized using Petri nets. Böhmer and Rinderle-Ma (2017), based on behavioral similarity, compared the multi-instance runs to be detected with historical execution records documented based on temporal relationships to predict low-frequency anomalies. This method focuses on multi-case executions on a global scale and demonstrates excellent performance in real logs with high concept drift. Table 1 shows the differences between MLGTAE and GAE, BINet, and GRASPED.

**Table 1:** Comparison of MLGTAE with related anomaly detection methods.

**Method**	**Core architecture**	**Input representation**	**Behavioral semantics handling**
GAE	Graph autoencoder	Directly-follows graph	Limited
Binet	Recurrent neural network	Sequential data	Indirect
GRASPED	GRU + autoencoder	Sequential data	Indirect
MLGTAE	Graph transformer + autoencoder	Multi-perspective trace graphs	Rich

## 3 Background knowledge

### 3.1 Relevant definitions

Definition 1 Event (Taymouri et al., 2021). Let *E* be the event domain, *A* represent the activity domain, and *T* denote the time domain. For any event *e*∈*E*, it can be expressed as *e* = (*eid, cid, a, t, b*_1_, ⋯ , *b*_*i*_), where *eid* is the event sequence number, *cid* is the sequence number of the activity in the corresponding case, *a* ∈ *A*, *t*∈*A* represents the activity executed by the event, t is the specific time when the activity is executed, and *b*_1_, ⋯ , *b*_*i*_ represents other additional attribute values.

Definition 2 Case, Trace, Event Log (Taymouri et al., 2021). A case is a specific instance containing multiple events, denoted as *c* = *e*_1_, *e*_2_, ⋯*e*_*n*_(*n*≥). A trace is the sequence of activities in a case, denoted as *trace* = < *a*_1_, *a*_2_, ⋯ , *e*_*n*_ >, where *a*_*n*_ = #_*activity*_(*e*_*n*_) represents the mapping of events to corresponding activities, and the occurrence times of adjacent activities are in ascending order, that is, *t*(*a*_*i*_) ≤ *t*(*a*_*i*+1_) and 1 ≤ *i* ≤ *n*. An event log is a set of multiple cases, denoted as *L* = {*c*_*i*_∈*S*|1 ≤ *i* ≤ *m*}, where *S* represents all possible cases, and represents the number of cases contained in the event log.

As shown in Table 2, it is a partial snippet of the hotel check-in log, which includes two case (*K, G*) and 13 events. Each event in the cases contains four attributes: event ID, case ID, activity name, and activity occurrence time. In the original log, there are additional data information similar to resources (event executor or object) to record the corresponding attributes of the activity execution.

**Table 2:** Fragment of hotel check-in event log.

**Event ID**	**Case ID**	**Activity**	**Timestamp**
...	...	...	...
576	K	New	2013-01-22 09:35:15
578	K	Advance order	2013-01-22 09:35:34
579	K	Fin	2013-04-23 20:00:40
580	K	Release	2013-04-24 00:24:39
581	K	Check order	2013-04-28 01:44:04
582	K	Billed	2013-08-03 19:03:37
583	G	New	2012-12-24 07:34:33
584	G	Advance order	2012-12-24 15:43:37
585	G	Advance order	2012-12-29 21:05:06
586	G	Fin	2012-12-30 15:26:29
587	G	Release	2012-12-31 06:31:16
588	G	Check order	2013-01-06 05:29:27
589	G	Billed	2013-04-03 23:34:05
...	...	...	...

Definition 3 Petri Net (Zhao et al., 2022). A Petri net can be represented as a triple, where *S* represents the set of places in the net, *T* represents the set of transitions in the net, and *F* represents the flow relationship between places and transitions, with the existence of *S*∪*T*≠∅, *S*∩*T* = ∅, and *F*∈(*S*×*T*)∪(*T*×*S*) rule relationships.

Definition 4 Identifier Net (Ma et al., 2021). An identifier net can be represented as a quadruple (*S, T, F, L*), where *L*:*S* is a mapping acting on places, and *L*:*S* → {0, 1, 2, ⋯ } represents the number of identifiers contained in the places.

**Figure 1:**
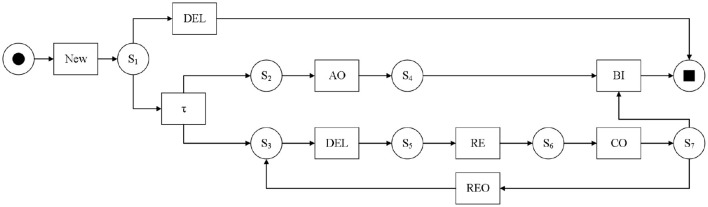
Petri net model of hotel check-in partial log.

As shown in Figure 1, it is the Petri net model of the hotel check-in part log based on Table 1. In the model, ADVANCE ORDER, RELEASE, CHECK ORDER, BILLED, REOPEN, and DELETE activities are abbreviated as AO, RE, CO, BI, REO, and DEL respectively. To ensure that the process model can fully represent the sequence of events in the real scenario, invisible transitions (τ) are usually present in the process model to guarantee its smooth operation. Each reasonable operation of the net generates a trace. For the above Petri net, < NEW, AO, FIN, RE, CO, BI> is a possible trace. In the entire net system in the figure, only place *S*_*I*_ has the marking *L*(*S*_*I*_) = 1, and the only transition that can be triggered currently is the NEW activity.

Definition 5 Behavioral Footprint (Weidlich and Van Der Werf, 2012). For the Petri net model *MP*, *AC* is a mapping function from transitions to activities, and for any pair of activities (*A, B*)∈(*AC*(*t*_1_) × *AC*(*t*_2_)), one of the following four behavioral footprint relationships always holds:

(1) If in the model, there is a path where activity *B* directly follows activity *A*, then *A* and *B* are said to have a direct successor relationship, denoted as *A*→*B*.

(2) If in the model, activities *A* and *B* have a direct successor relationship, then simultaneously *B* and *A* are said to have a direct predecessor relationship, denoted as *B*←*A*.

(3) If in the model, there is a path where activity *A* directly follows activity *B* and there is also a path where activity *B* directly follows activity *A*, then *A* and *B* are said to have a concurrent relationship, denoted as *A*∥*B*.

(4) If in the model, there is no direct path relationship between activity *A* and activity [[Mathtype-mtef1-eqn-41.mtf]], then *B* and *A* are said to have an exclusive relationship, denoted as *A#B*. The collection of these four relationships is called the behavioral footprint of the Petri net model, denoted as *Footpr*int = { → , ←, ∥, #}.

### 3.2 Graph convolutional neural network

Graph Convolutional Network (GCN) (Zhang et al., 2022) can perform convolution operations on graph data to learn the information of the current node and its neighboring nodes. The specific formula is as follows:


$$\begin{array}{l} X^{\left(l+1\right)}=\sigma \left(AX^{\left(l\right)}W^{\left(l\right)}\right) \end{array}$$ (1)

Here, *A* is the adjacency matrix, representing the connection relationship between nodes in the graph, *X*^(*l*)^ represents the feature matrix of the upper-level nodes, *W*^(*l*)^ and σ respectively denote the learnable weights and nonlinear activation functions. However, this is a basic formula, which lacks consideration of the self-features during information aggregation. Therefore, the identity matrix is added to solve this problem. Moreover, since the degree of each node is different, the feature propagation may change due to this factor. To address this issue, the degree matrix needs to be introduced and normalized. The improved formula is as follows:


$$\begin{array}{l} X^{\left(l+1\right)}=\sigma \left({\tilde{D}}^{-\frac{1}{2}}Ã{\tilde{D}}^{-\frac{1}{2}}X^{\left(l\right)}W^{\left(l\right)}\right) \end{array}$$ (2)

Among them, ${\tilde{D}}^{-\frac{1}{2}}Ã{\tilde{D}}^{-\frac{1}{2}}$ is the normalized result, σ represents the activation function, $\tilde{D}$ is the degree matrix, and Ã = (*A*+*I*) is the unit matrix.

### 3.3 Transformer

**Figure 2:**
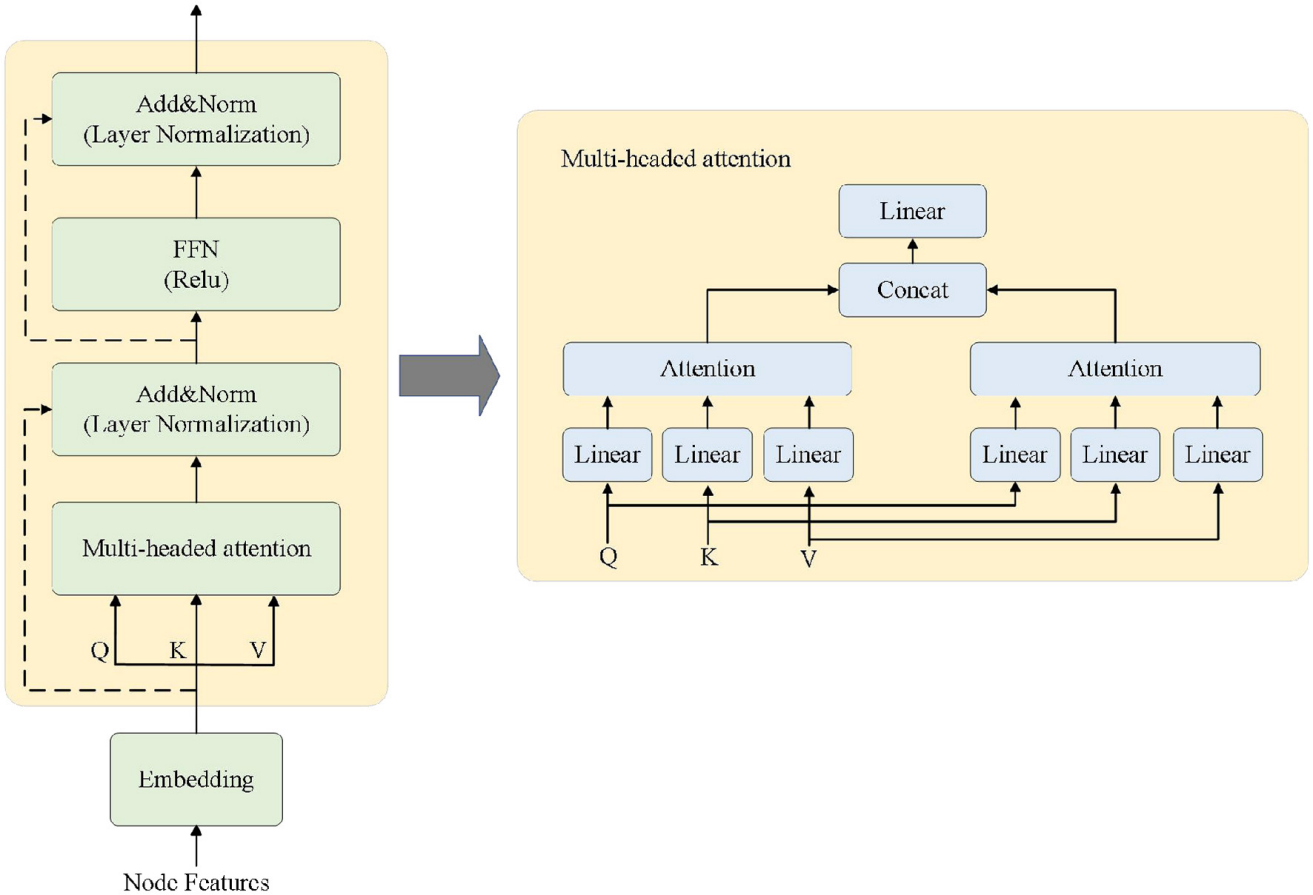
Transformer encoder and multi-head attention mechanism.

Transformer (Huang et al., 2020) is a deep learning framework mainly consisting of key sub-layers such as multi-head attention mechanism, feed-forward neural network, and residual connection, as shown in Figure 2. This paper mainly focuses on the encoder part of the Transformer model, which is an important component of the proposed encoder for anomaly detection framework. The Transformer model can consider all the information of the input sequence globally and assign weights to different positions based on the importance of the information, making the processing of long-distance and long-sequence information a reality.

The right side of Figure 2 shows the specific architecture of the multi-head attention mechanism. The multi-head attention mechanism can capture the dependency patterns among multiple attributes, which is highly suitable for multi-attribute and multi-perspective anomaly detection tasks in business processes. The detailed definition of multi-head attention is as follows:


$$\begin{array}{l} Attention\left(Q,K,V\right)=soft\;max\left(\frac{QK^{T}}{\sqrt{d_{k}}}\right)V \end{array}$$ (3)

First, the attention score is calculated by using Formula 3 through the query *Q* and key-value pairs *K*−*V*. Here, *Q*, *K*, and *V* are obtained by multiplying the input vector with the parameter matrix. The role of $\sqrt{d_{k}}$ is to ensure the stability of the gradient. *soft*max normalizes the obtained values. The entire attention function finds the most relevant results through mapping.

Then, since multi-head attention uses multiple heads to learn correlations in different dimensions, it is necessary to perform weighted calculations on the attention values of each head:


$$\begin{array}{l} h_{i}=Attention\left(QW_{i}^{Q},KW_{i}^{K},VW_{i}^{V}\right) \end{array}$$ (4)

Finally, the calculation results of multiple heads are combined and the final result is obtained through a linear transformation.


$$\begin{array}{l} MH=Concat\left(h_{1},h_{2},\cdots \;,h_{i}\right)w \end{array}$$ (5)

This processing approach enables deep-level interaction among multiple attributes and utilizes a multi-head approach to achieve multi-perspective, multi-perspective mutual influence as well as parallel computing to enhance processing efficiency.

### 3.4 Additive attention mechanism

Additive Attention (Brauwers and Frasincar, 2023) is the foundation for achieving multi-perspective attribute interaction in the architecture. It focuses on the parts of the data that have a greater impact on the current task by inputting the hidden outputs of multiple encoders. Additive Attention uses a weight matrix to transform the dimensions of the encoder output and the current decoder hidden state, then concatenates the two and processes them with a nonlinear activation function to calculate the attention score. The specific calculation formula is as follows:

Concatenation of the weight matrix and the input vector:


$$\begin{array}{l} W_{c}=Concat\left(W_{q},W_{k}\right) \end{array}$$ (6)

$$\begin{array}{l} h_{k}=Concat\left(h_{i-1},s_{i}\right) \end{array}$$ (7)

Calculation of attention scores:


$$\begin{array}{l} Score_{i}=V^{T}\text{tanh}\left(W_{c}h_{k}\right) \end{array}$$ (8)

Equivalent to:


$$\begin{array}{l} Score_{i}=V^{T}\text{tanh}\left(W_{q}h_{i-1}+W_{k}s_{i}\right) \end{array}$$ (9)

Normalization:


$$\begin{array}{l} Score=soft\text{ max}\left(Score_{0},Score_{1},\cdots \;,Score_{i-1}\right) \end{array}$$ (10)

Among them, *V*, *W*_*q*_, and *W*_*k*_ are all learnable parameters. *h*_*i*−1_ is the hidden vector output by the encoder module, and *s*_*i*_ is the hidden vector of the decoder at the current time. tanh is then used as the activation function to obtain the attention score. Finally, the *soft*max function is used for normalization processing. Additive attention mainly achieves the allocation of weights by calculating the similarity between the query and the key. The main function of formulas 6 and 7 is to concatenate the weight matrix and the input vector, while the main function of formulas 8 and 9 is to obtain the attention score by calculating the similarity. The function of formula 10 is to normalize the weights so that their sum is 1.

### 3.5 LSTM

**Figure 3:**
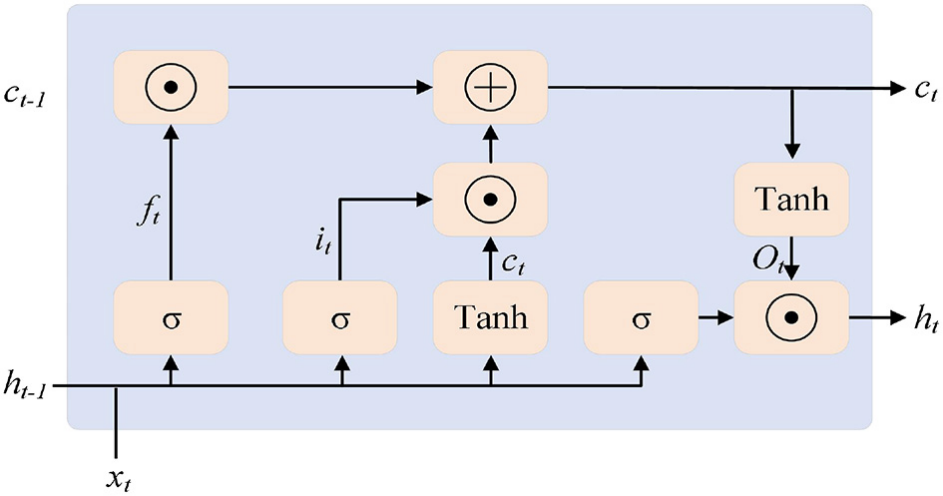
LSTM neural unit.

The long short-term memory (LSTM) network (Lindemann et al., 2021) serves as the decoder part of the proposed framework. By stacking multiple LSTMs, it provides decoding results for the anomaly detection objective. LSTM is a variant of the recurrent neural network (RNN). As the RNN model processes sequences, it may encounter problems such as vanishing or exploding gradients. To address this issue, the LSTM model introduces three gated logics: the forget gate, the input gate, and the output gate (corresponding to *f*_*t*_, *i*_*t*_, and *o*_*t*_ in Figure 3). The forget gate processes the existing information from the past, retaining the information that has a significant impact on the next moment and ignoring the unimportant information. The input gate combines the retained information from the previous stage with the new input information. The output gate mainly integrates these pieces of information. In the figure, *c*_*t*_ represents the complete memory, and *h*_*t*_ represents the hidden output for the next moment, indicating which information is used for the next moment.

## 4 Method

This section will introduce the proposed anomaly detection method in detail through three parts. The first part processes the event logs, constructs a graph structure based on activities for each case with behavioral relationships as constraints, and represents other attribute features in the form of node features to build multi-perspective traces as the input of the deep learning model. The second part mainly introduces the architecture of the deep learning model used in this paper in detail. The third part introduces the specific calculation method of anomaly scores, which is mainly divided into two levels: attribute level and trace level.

**Figure 4:**
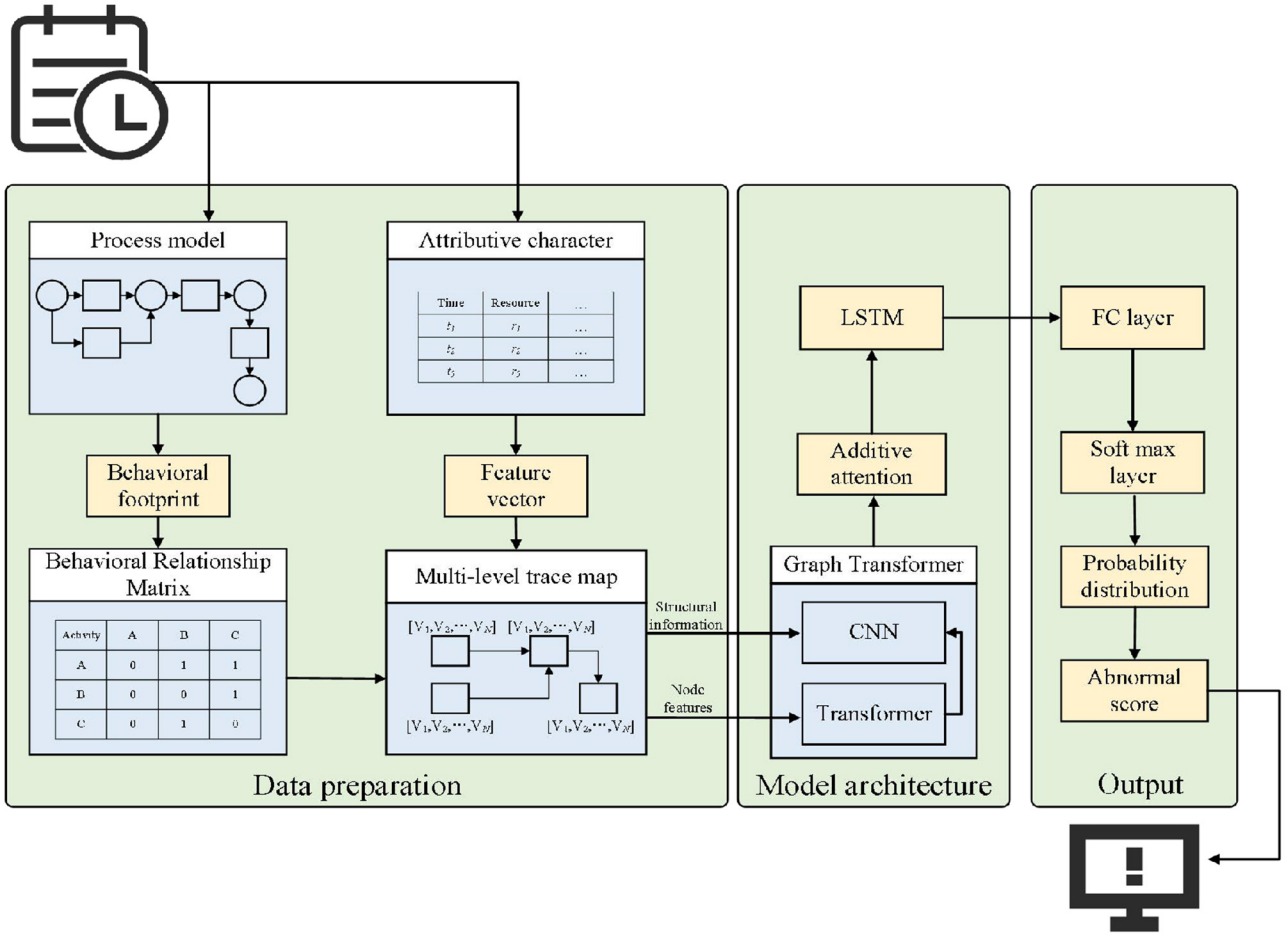
Overall framework.

As shown in Figure 4, the overall framework of the proposed method is presented. To incorporate fine-grained behavioral information into the anomaly detection process, MLGTAE first uses the Inductive Miner algorithm to mine the Petri net model from the event log, and then constructs the behavioral relationship matrix through the behavioral footprint theory to standardize the construction of the trace graph. Further, it extracts the attributes corresponding to the current trace and assigns them as node features to the graph structure to generate multi-perspective trace graphs. Next, the multi-perspective trace graphs are input into the encoder-decoder architecture of the deep learning model. The encoder's main component is the Graph Transformer, which combines the advantages of GCN and Transformer, enabling the model to handle graph data while having the ability to process long sequences. Moreover, the introduction of Transformer allows for the acquisition of global information perception. The decoder's key components are additive attention and multi-layer LSTM, whose main function is to interact and decode multi-perspective information. Finally, anomalies are identified by calculating the anomaly scores of different attributes.

### 4.1 Data preparation

#### 4.1.1 Behavioral relationship matrix

This subsection mainly introduces the construction of the Petri net behavior matrix and the construction method of the input for the anomaly detection model, that is, the construction of multi-perspective trace graphs. Behavior relationship matrix.

Traditional methods using sequences ignore behavioral information. While graph neural networks (GNNs) have been proposed, representing traces via a directly-follows graph remains problematic. In the context of anomaly detection, if only the direct-following relationships between activities are focused on, it may be reasonable for the current trace, but when the perspective is broadened to the entire log, there are certain deficiencies. To solve this problem, this paper constructs the local trace graph by extracting the behavior relationship matrix from the process model, thereby enhancing the focus on behavioral information during the learning process. The specific steps are shown in Algorithm 1.

Algorithm 1 **Construction of behavior relation matrix**

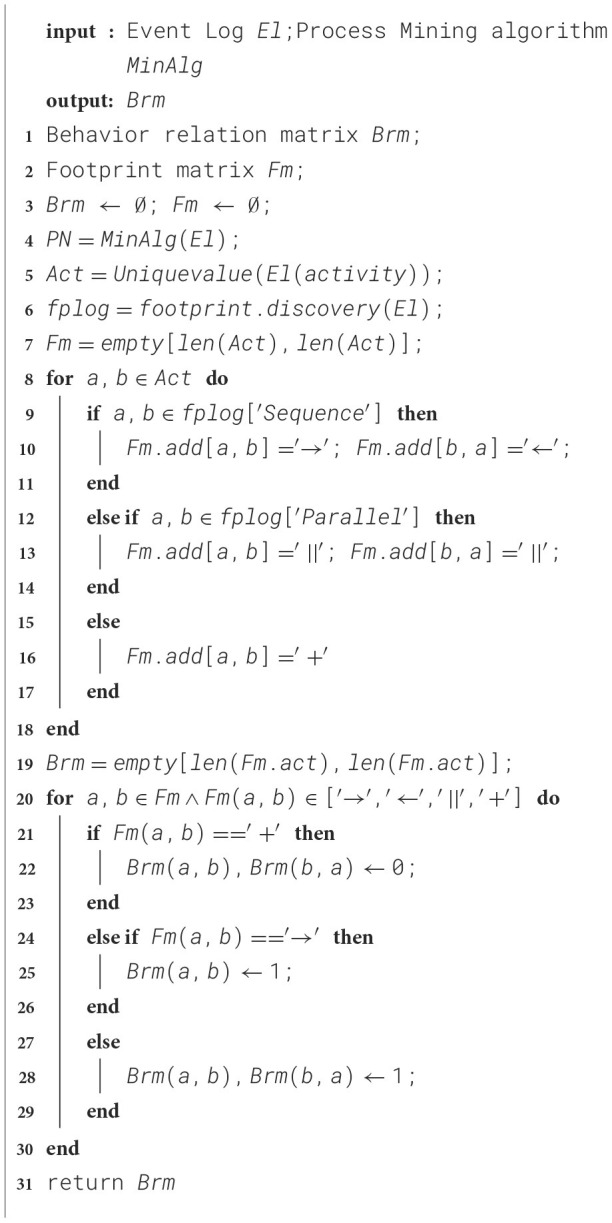

Algorithm 1 takes the event log *El* and the available process mining algorithm as input. Lines 1–3 of the algorithm define the behavior relationship matrix *Brm* and the footprint matrix *Fm* and initialize them. Lines 4–6 of the algorithm mine the Petri net from the event log, further extract all unique activities from the event log, and extract the behavioral footprint from the obtained process model.

Lines 7 to 18 of the algorithm mainly construct the behavioral footprint matrix of the current process model, which includes four types of behavioral relationships: direct successor relationship (‘ → '), direct predecessor relationship (‘←'), parallel relationship (‘∥'), and exclusive relationship (‘+').

The behavioral relationship between activities has been obtained. Next, the footprint matrix needs to be converted into a numerical matrix to standardize the structure of the trace graph. Lines 19 to 30 determine the behavioral relationship between activity pairs in the footprint matrix. If the relationship is exclusive, the corresponding position is filled with 0. If it is a direct successor relationship, the corresponding position is filled with 1. The same applies to parallel relationships, where the corresponding position is also filled with 1. The final output is a behavioral relationship matrix containing only 0s and 1s.

**Figure 5:**
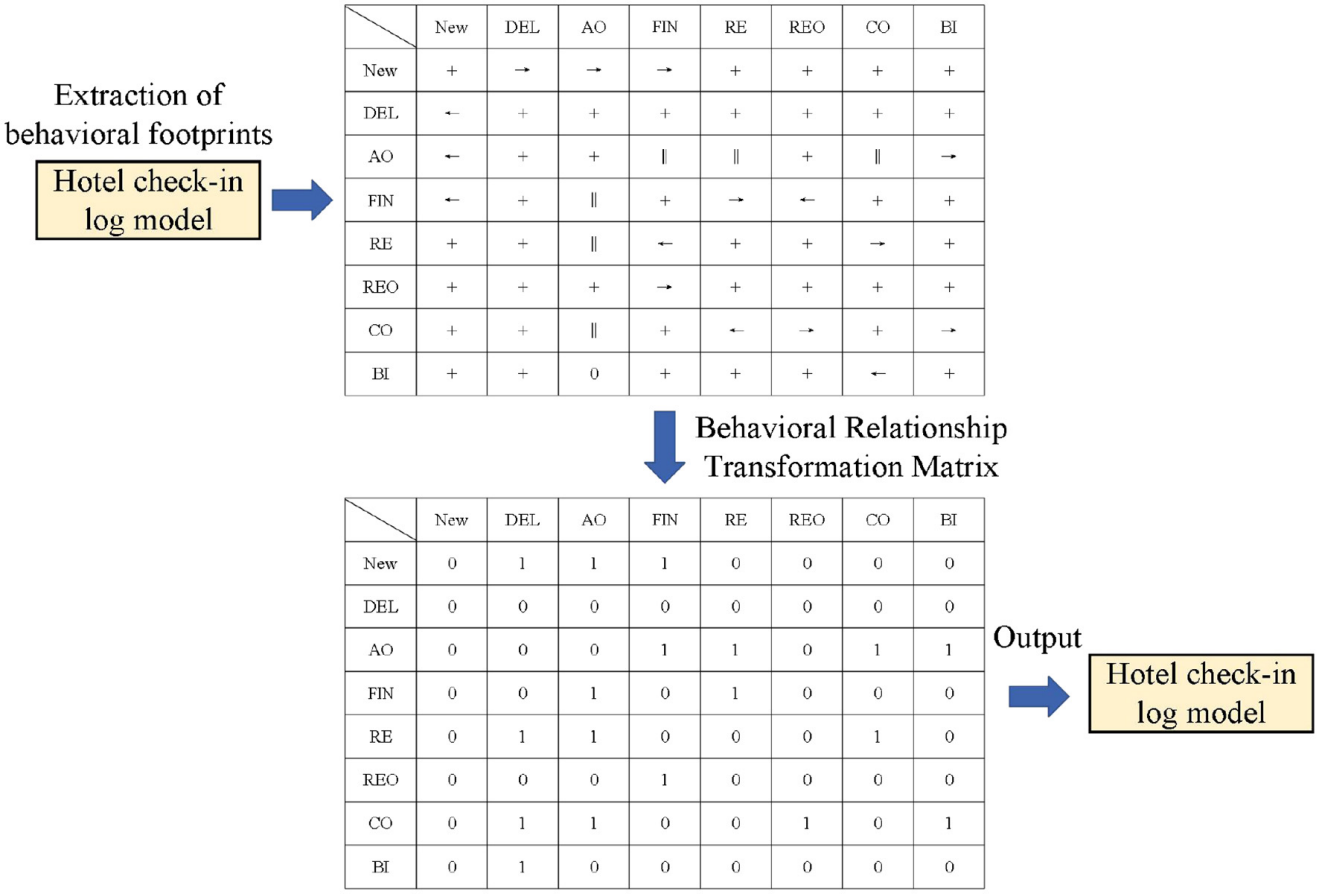
Behavioral relationship matrix.

As shown in Figure 5, the process of constructing the behavioral relationship matrix for the Petri model of the medical billing partial log is as follows: First, the behavioral footprints are extracted from the process model to construct the footprint matrix. It can be seen that the footprint matrix contains four types of behavioral footprint relationships, such as the parallel relationship between activity AO and activities FIN, RE, and CO. Then, the footprint matrix is transformed into a behavioral relationship matrix, which assigns 0 or 1 to the possible relationships between activities to standardize the construction of the trace graph.

Based on the behavioral relationship matrix, it can be seen that there is a concurrent relationship between activities CD and FIN. For the For Trace K: < NEW, AO, FIN, RE, CO, BI>, a directly-follows graph would enforce a sequential relationship between AO and FIN, which violates their actual concurrent relationship. Our behavioral matrix normalization method retains this correct behavioral information. This is the construction of the activity-level graph. For other attributes, by retaining the structure of the activity graph while changing the node characteristics (taking the current attribute as the node characteristic), the corresponding attribute graph can be obtained.

#### 4.1.2 Construction of multi-perspective trace maps

The multi-perspective trace graph serves as a bridge to effectively combine process behavior and data. This method constructs a trace-specific multi-perspective graph based on the behavioral footprint theory to improve the integration of behavioral relationships and data interaction. The specific steps are shown in Algorithm 2.

Algorithm 2 **Constructing multi-level graph input**

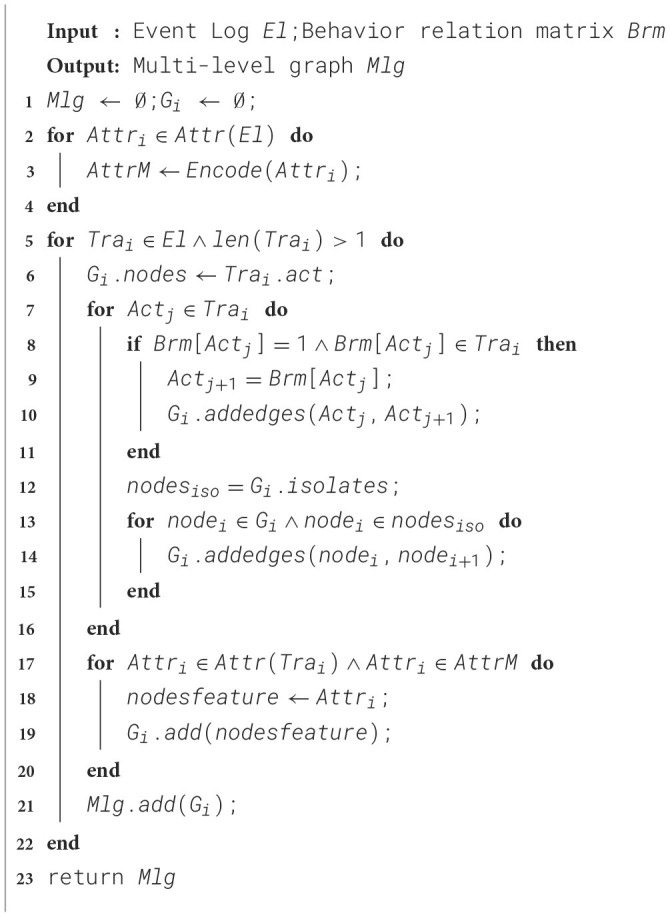

Algorithm 2 takes the event log *El* and the behavioral relationship matrix *Brm* obtained in the previous step as input and outputs a multi-perspective attribute graph. The first line of the algorithm initializes the multi-perspective graph set *M*lg and the attribute graph *G*_*i*_ of the current trace. Lines 2 to 4 are a process of attribute encoding, which requires traversing each attribute contained in the trace and encoding it. Categorical attributes, numerical attributes, and time attributes are encoded in the form of feature vectors. The encoding operation will be explained in detail later.

Lines 5 to 16 of the algorithm, for all traces in the event log with a length greater than 1, first add all the unique activities of the current trace to the trace graph as graph nodes. Then, for each activity in the trace, find the activities corresponding to it in the behavioral relationship matrix with a value of 1 and add directed connection lines. These two activities are in a parallel or direct successor relationship. At this point, the construction of the trace graph fully considers the global behavioral relationship. Finally, to ensure the connectivity of the graph, isolated nodes in the graph are judged. If there are isolated nodes, they are connected to the possible next activity to ensure the connectivity of the graph. Lines 17 to 23, the main purpose is to assign the corresponding attributes of the activities contained in the current trace to the graph nodes to construct a multi-perspective trace graph. For each attribute, the vector form after encoding is assigned to the corresponding node as a feature, obtaining a multi-perspective graph about the current trace. Finally, the set of graphs *M*lg is returned.

#### 4.1.3 Feature encoding

The encoding process of attributes is described based on the Trace K in Table 1. The business process includes categorical attributes, numerical data, and time attributes. For categorical data, one-hot encoding is used to achieve the encoding. This encoding method mainly defines the dimension through the unique values of the attribute. For example, Trace K has 6 activities, and only for this trace, it will be encoded as a feature matrix of [6,6]. All categorical attributes are encoded in a similar way. However, for time attributes, since they are continuous attributes, they need to be discretized. In this paper, the equal-frequency binning method is used to convert them into categorical attributes and then encoded. For example, for a set of data [1, 10, 15, 20, 35, 50, 70], it is equally frequency binned into 2 bins, so this set of data is mapped to [1, 1, 1, 1, 2, 2, 2], and then the bin number corresponding to the time is encoded. During the encoding process, missing categorical attributes are filled with “miss”, and missing time attributes are filled with the initial time.

#### 4.1.4 Model architecture

**Figure 6:**
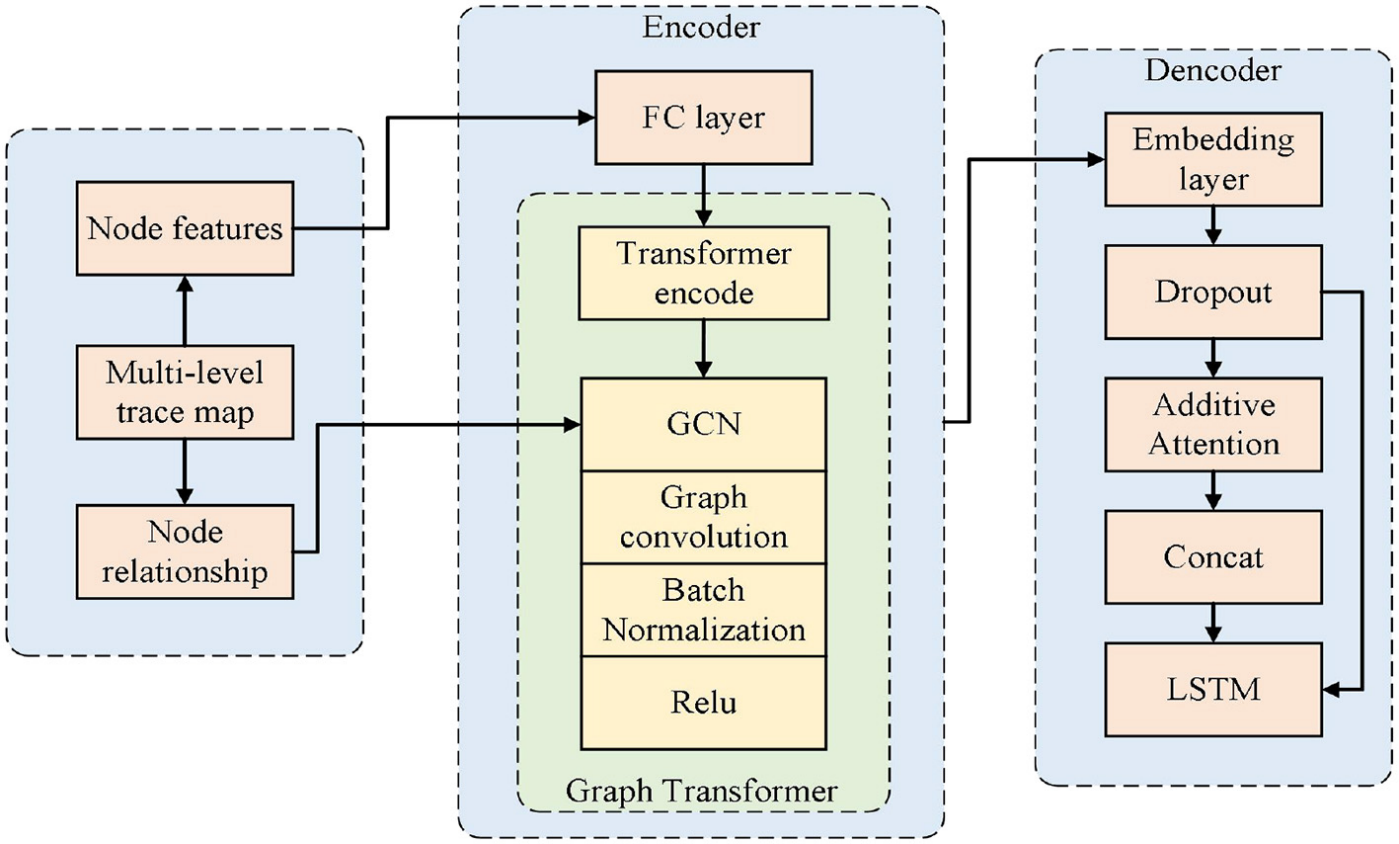
Specific encoder-decoder architecture.

As shown in Figure 6, on the left is the encoder part of the proposed detection model. Firstly, the node features (i.e., attributes) of the multi-perspective trace graph are input into a fully connected layer to match the input dimension with the hidden layer dimension. Then, a Transformer encoder is used to process the node features. Based on the attention mechanism of Transformer, it can provide a global perception at the attribute level and enable the model architecture to handle long sequences, providing support for large-scale business scenarios. Finally, the output of the Transformer and the edge relationships between nodes are input into GCN to achieve the representation of the trace graph. Graph Transformer combines the characteristics of Transformer and GCN, enabling the model to have the ability to represent graphs while paying attention to both global and local information, thereby improving the performance of anomaly detection.

The right side of Figure 6 shows the decoder part of the detection model. Firstly, the output of the encoder is passed to the decoder, and the embedding layer and Dropout layer are used to match the dimensions and prevent overfitting. Then, through additive attention, the interaction effects among different attributes are learned. Finally, the information from the attention output and the hidden information from the previous step of the decoder are concatenated and input into a multi-layer LSTM model for decoding. The output of the LSTM is then used to calculate the anomaly score, and the final anomaly detection result is obtained.

During the model training process, cross-entropy is adopted as the loss function, as shown in Formula 11. This function measures the loss by judging the distance from the expected value. The dimension of the hidden layer is 256, and the number of layers for both the encoder and decoder is 2. The initial learning rate for training is 0.001, but a learning rate optimization strategy is set during the training process. When the loss function does not decrease within a certain number of time steps, the learning rate will be reduced according to the given parameters.


$$\begin{array}{l} CE\left(y,y\right)=\underset{i}{\sum }y\left(i\right)log\left(y\left(i\right)\right) \end{array}$$ (11)

#### 4.1.5 Abnormality identification

When the trace to be detected, which contains multi-perspective information, is input into the proposed method, a corresponding probability distribution will be generated. Generally, the probability of an anomaly occurring is much smaller than that of a normal event. This paper refers to References (Nolle et al., 2022) and (Guan et al., 2023a,b) to define the anomaly score and identify anomalies in the trace from multiple perspectives. The anomaly score is defined as the sum of all probabilities greater than the current activity or attribute in the probability distribution, and its formula is as follows:


$$\begin{array}{l} AS\left(p_{j}\right)=\underset{p_{i}>p_{j}}{\sum }p_{i} \end{array}$$ (12)

Among them, *p*_*j*_ is the attribute or activity for which the abnormal score needs to be calculated, and its result is the sum of the probabilities in the probability distribution that are greater than the probability *p*_*i*_ of *p*_*j*_.

To illustrate the calculation method of the abnormal score, for the Trace K in Table 1, with the activity sequence < New, AO, FIN, RE, CO, BI>, assuming its corresponding probability distribution is (0.05, 0.10, 0.35, 0.25, 0.12, 0.13), the current position's actual activity is CO, with a corresponding probability of 0.12, and the abnormal score is the sum of the probabilities greater than it, which is 0.73. This step is the same operation for data attributes.

In order to identify anomalies, an abnormal threshold needs to be defined to map the calculated abnormal score to the abnormal-normal interval, and the specific formula is as follows:


$$\pi \left(AS,t\right)=L_{AS}=\{\begin{array}{c} 1\;if\;AS>t\\ 0\text{ otherwise} \end{array}$$ (13)

Here, π represents the mapping relationship, and *t* is the set threshold. In this paper, *t* is a fixed hyperparameter determined empirically, not learned through model training. In our experiments, we select the optimal threshold by evaluating the F1-Score on a validation set (a subset of the training data) to balance precision and recall. The example of $\tau = 0.7$ in the following text is for illustrative purposes only. For a certain activity or attribute, if its anomaly score is greater than the set threshold, it is mapped to label 1, meaning that this value is abnormal. Conversely, it is mapped to label 0, indicating that this value is reasonable.

For the above Trace K, if the current set threshold is 0.7, and the calculated anomaly score for the CO activity is 0.73, it is determined to be an abnormal activity. By calculating the anomaly score, fine-grained anomalies at the trace and attribute levels can be located.

## 5 Experimental evaluation

This section will conduct a detailed evaluation of MLGTAE. Firstly, it mainly introduces the relevant settings for the evaluation. Then, it presents the datasets used for the evaluation. Finally, it discusses the results obtained by the proposed method and the comparison methods.

### 5.1 Experimental setup

To verify the superiority of MLGTAE in anomaly detection, it will be compared with several advanced methods including Naive (Bezerra and Wainer, 2013), Sampling (Bezerra and Wainer, 2013), GAE (Huo et al., 2021), DAE (Nolle et al., 2018), and Binet (Nolle et al., 2022). Among them, Naive directly defines anomalies based on occurrence frequency, which may lead to incorrect detection and is a relatively strict detection strategy; Sampling, based on the characteristic that anomalies are infrequent, uses sampling to obtain a portion of event logs to construct a process model, and anything beyond the model's boundaries is defined as an anomaly; GAE utilizes an autoencoder to encode log information into graph data as input for anomaly determination; DAE is an anomaly detection method that employs a denoising autoencoder, which requires no prior knowledge and can train models on noisy data; Binet is an anomaly detection framework that achieves attribute-level anomaly detection by predicting the next event.

### 5.2 Exception insertion and exception types

**Figure 7:**
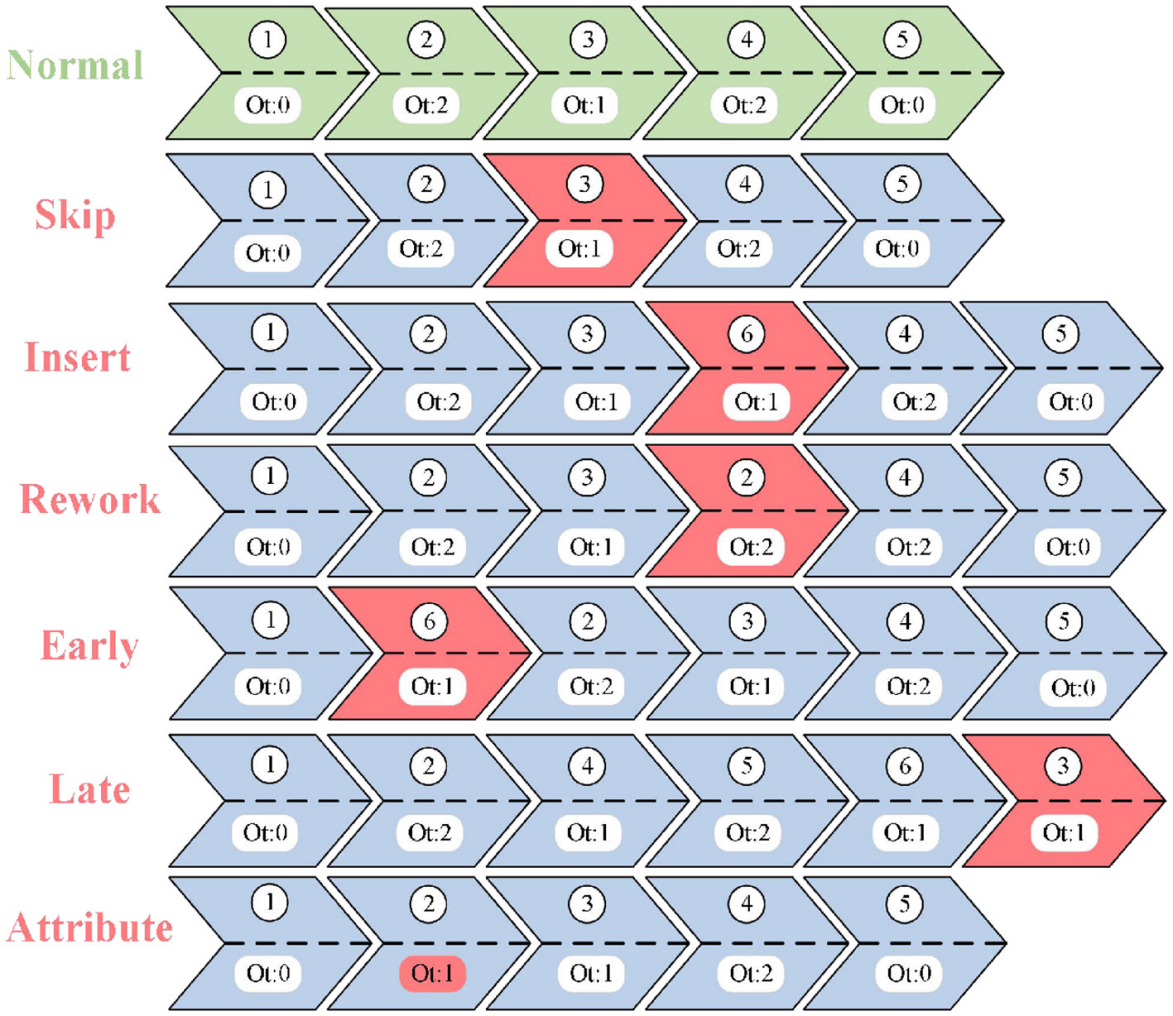
Abnormal types.

To simulate abnormal situations, it is necessary to insert artificially defined anomalies into the normal event logs to verify the effectiveness of MLGTAE. In the research of business process anomaly detection, to simulate typical deviations in real scenarios and comprehensively evaluate the model performance, this paper, referring to existing References (Nolle et al., 2022) and (Krajsic and Franczyk, 2021), defines six types of artificial anomalies, including jump, insertion, reconfiguration, advancement, delay, and attribute anomalies. These anomalies cover three key dimensions: control flow, time, and data attributes. Jump, insertion, and reconfiguration anomalies mainly simulate sequence disorder, omission, or redundancy in activity sequences; advancement and delay anomalies focus on the issues of premature or delayed execution at the time level; attribute anomalies target unreasonable values of data attributes such as resources and numerical values. The selection of these six types of anomalies aims to comprehensively reflect the multi-dimensional and fine-grained deviations that may occur in business processes, thereby effectively validating the comprehensive capabilities of the proposed method in trajectory-level and attribute-level anomaly detection. Figure 7 shows the different abnormal trajectories obtained by applying these six anomalies to normal trajectories. Among them, jump, insertion, reconstruction, advancement, and delay belong to trajectory anomalies, while attribute anomalies are reflected in attribute values. In Figure 7, green represents normal business process trajectories, blue represents business process trajectories with anomalies, and red represents the abnormal parts. The sequence number indicates the execution order of the business process trajectory, and Ot represents the attribute value contained in the event.

### 5.3 Dataset introduction and evaluation metrics

#### 5.3.1 Datasets and evaluation metrics

In the experiment, four real event logs will be used to evaluate MLGTAE. Among them, Hotel Billing is a business process about hotel room bill processing, Sepsis (Chicco and Jurman, 2020) is the detection process of sepsis, including activities such as detection and triage, Receipt is the approval process of licenses, including activities such as receiving applications and approving applications, and BPIC13_O (Steeman, 2013) is enterprise management data. To verify the anomaly detection effect of the method, specific labels were assigned during the process of inserting anomalies. MLGTAE transformed the anomaly detection task into a classification task. Therefore, we adopted three commonly used metrics in machine learning classification tasks, namely Precision (Juba and Le, 2019), Recall (Juba and Le, 2019), and F1-Score (Juba and Le, 2019).

We briefly analyze the computational complexity of MLGTAE. Let *N* be the maximum trace length (number of nodes), [[Mathtype-mtef1-eqn-95.mtf]] be the number of edges in the trace graph, and *D* be the hidden dimension. The GCN layer's complexity is approximately *O*(*E*▪*D*). The Graph Transformer's self-attention mechanism is the primary bottleneck, with a complexity of *O*(*N*^2^▪*D*). The decoder, based on a multi-layer LSTM of length $N$, has a complexity of *O*(*N*▪*D*^2^). Therefore, the overall complexity per trace during training is dominated by the Graph Transformer, resulting in *O*(*N*^2^▪*D*+*N*▪*D*^2^). In practice, since *N* (trace length) is generally much smaller than the total number of traces, the model remains computationally feasible.

### 5.4 Results discussion

The results discussion section conducts experiments on multiple real datasets and answers the following questions: (1) How do the results of MLGTAE compare with other advanced methods in terms of anomaly detection metrics? (2) Does the trace graph construction method guided by behavioral relationships achieve better results than the direct following graph method? (3) Is the proposed anomaly detection model affected by hyperparameters, and in which hyperparameters does it show robustness?

#### 5.4.1 Comparative experiments

**Figure 8:**
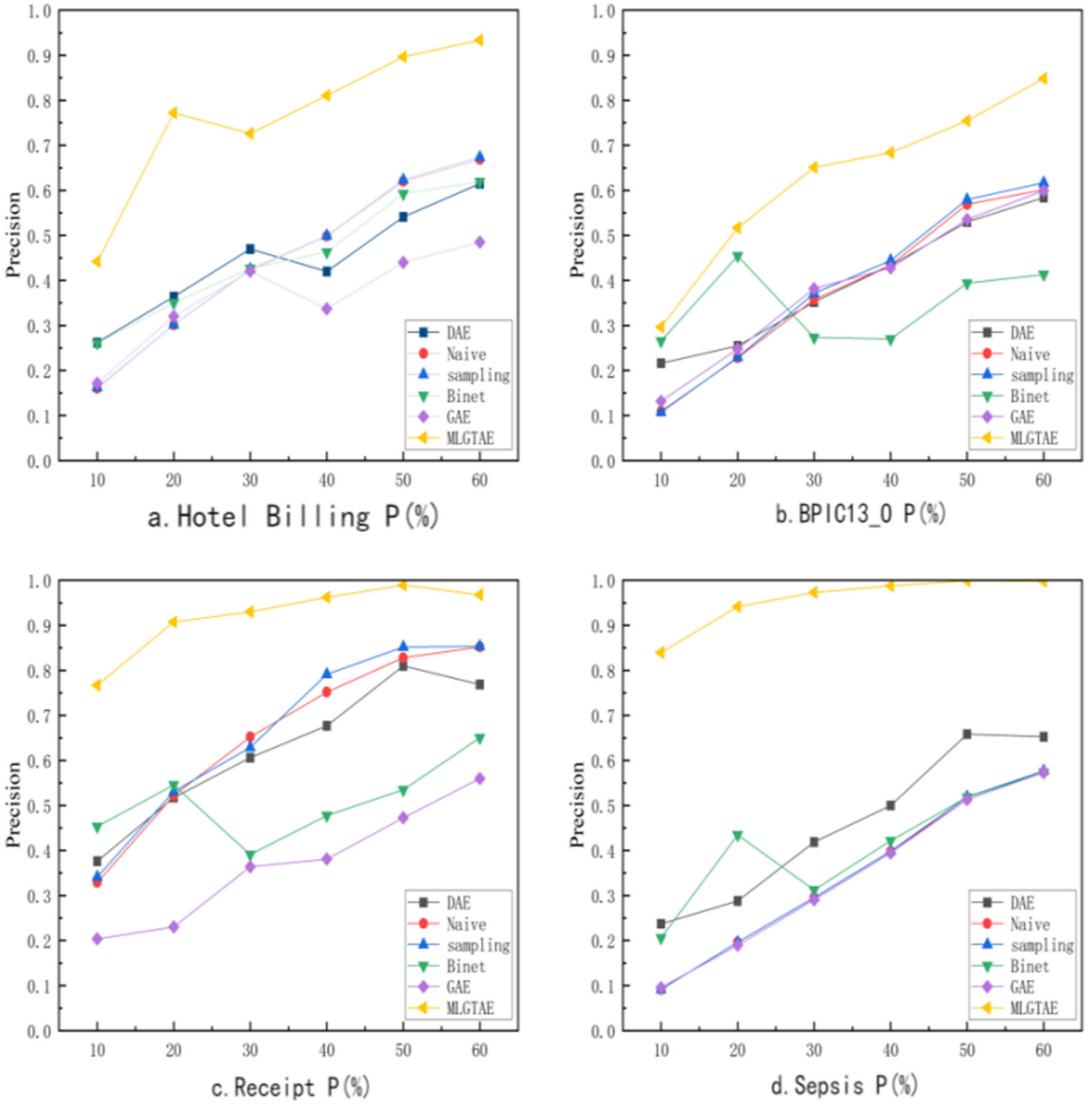
Anomaly detection accuracy of different methods (trace level).

This subsection will address question (1). As shown in Figure 8, it presents the precision results of anomaly detection under different methods and varying anomaly insertion ratios. The range of anomaly insertion ratio (P) considered in the figure is from 10% to 60%. In a typical environment, anomalies are usually low-frequency instances. However, to cover a broader range of scenarios, 60% is set as the maximum anomaly insertion ratio. The figure mainly consists of four subplots, each showing the precision results at the trace level on different datasets.

It can be observed that MLGTAE achieves good results under different abnormal insertion ratios in the four subgraphs. Moreover, in the Hotel Billing and Sepsis datasets, the highest accuracy reaches above 0.95. Meanwhile, GAE, which takes graph encoding as input, performs less ideally on these datasets, especially on the Hotel Billing and Sepsis datasets. This is because these two datasets have more activity types, meaning they have more complex structural relationships. However, conventional graph construction methods cannot incorporate this behavioral information.

Table 3 shows the F1-score results obtained at the trace level by different methods on the Receipt and BPIC13_O datasets. The better results are bolded. It can be observed that on these two datasets, MLGTAE achieved the best F1-score at any anomaly insertion ratio. In summary, MLGTAE has achieved good results at the trace level, whether on datasets with fewer activities (BPIC_13O) or on datasets with more activities (Sepsis).

**Table 3:** F1-scores of anomaly detection by different methods (trace level).

**Method proportion**	**DAE**	**Naive**	**Sampling**	**Binet**	**GAE**	**MLGTAE**
Hotel billing (10)	0.475	0.464	0.476	0.366	0.252	0.788
Hotel billing (20)	0.615	0.627	0.632	0.517	0.323	0.885
Hotel billing (30)	0.713	0.733	0.718	0.541	0.476	0.935
Hotel billing (40)	0.772	0.790	0.804	0.604	0.548	0.961
Hotel billing (50)	0.828	0.836	0.832	0.686	0.642	0.9740.983
Hotel billing (60)	0.845	0.852	0.851	0.769	0.717	0.935
BPIC3_O (10)	0.258	0.190	0.188	0.262	0.202	0.444
BPIC3_O (20)	0.343	0.353	0.354	0.382	0.344	0.641
BPIC3_O (30)	0.467	0.494	0.507	0.384	0.428	0.759
BPIC3_O (40)	0.538	0.562	0.567	0.413	0.449	0.812
BPIC3_O (50)	0.628	0.677	0.678	0.552	0.563	0.860
BPIC3_O (60)	0.662	0.697	0.702	0.577	0.543	0.918

**Figure 9:**
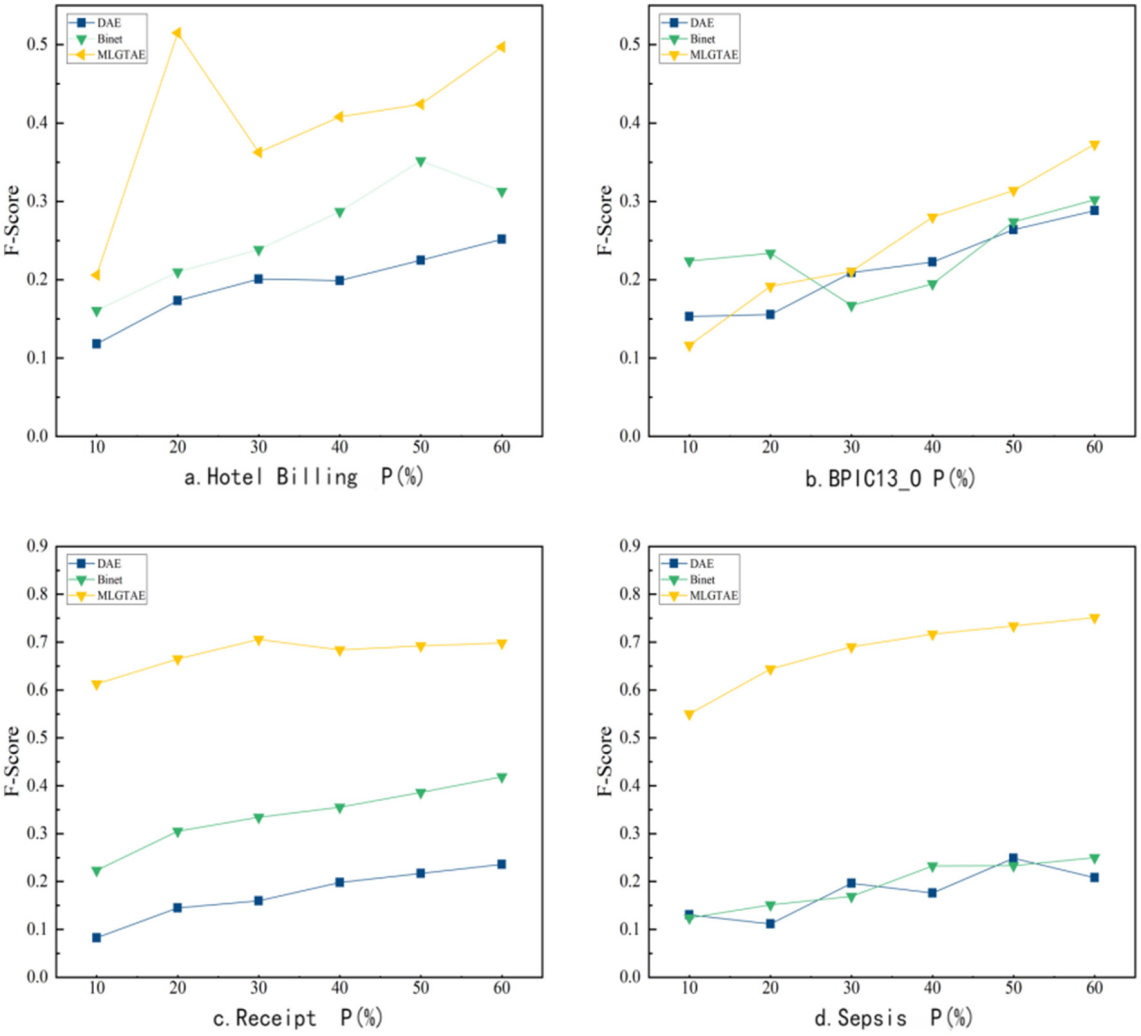
F1-scores of anomaly detection by different methods (attribute level).

Figure 9 shows the comparison results of F1-scores of MLGTAE and other methods at the attribute level. It can be observed that MLGTAE is generally superior to other methods. Meanwhile, it can be seen that Binet's anomaly detection performance at the attribute level is second only to MLGTAE, demonstrating the advantage of the deep model architecture in handling multiple features. At the attribute level, the performance of the DAE method is relatively poor, which indicates that the introduction of Transformer has enabled our method to achieve better detection results in multiple attributes.

Table 4 mainly presents the anomaly detection performance of MLGTAE on the datasets Billing and BPIC20_P. These two datasets have a larger number of cases and time points, meaning that larger graphs will be obtained when constructing graph inputs. We use these two datasets to test the learning ability of MLGTAE on large-scale graph data. As shown in the table, on the Billing dataset, the trace-level detection score reached 0.95 and the attribute anomaly score was 0.69. On the BPIC20_P dataset, the trace-level detection score reached 0.99 and the attribute anomaly score was 0.81. This indicates that MLGTAE still performs well on large-scale data.

**Table 4:** MLGTAE detection results (F1-score) on hotel billing and BPIC20_P datasets.

**Data level**	**Billing (10)**	**Billing (20)**	**Billing (30)**	**Billing (40)**	**Billing (50)**	**Billing (60)**
Trace	0.627	0.804	0.887	0.923	**0.934**	**0.950**
Attribute	0.208	0.300	0.684	0.687	**0.694**	0.691
**Datalevel**	**BPIC20_P (10)**	**BPIC20_P (20)**	**BPIC20_P (30)**	**BPIC20_P (40)**	**BPIC20_P (50)**	**BPIC20_P (60)**
						
Trace	0.887	0.953	0.979	0.991	**0.995**	**0.997**
Attribute	0.754	0.782	0.796	0.807	**0.814**	**0.812**

The bold values indicate the outstanding results in the experiment.

#### 5.4.2 Ablation experiment

**Figure 10:**
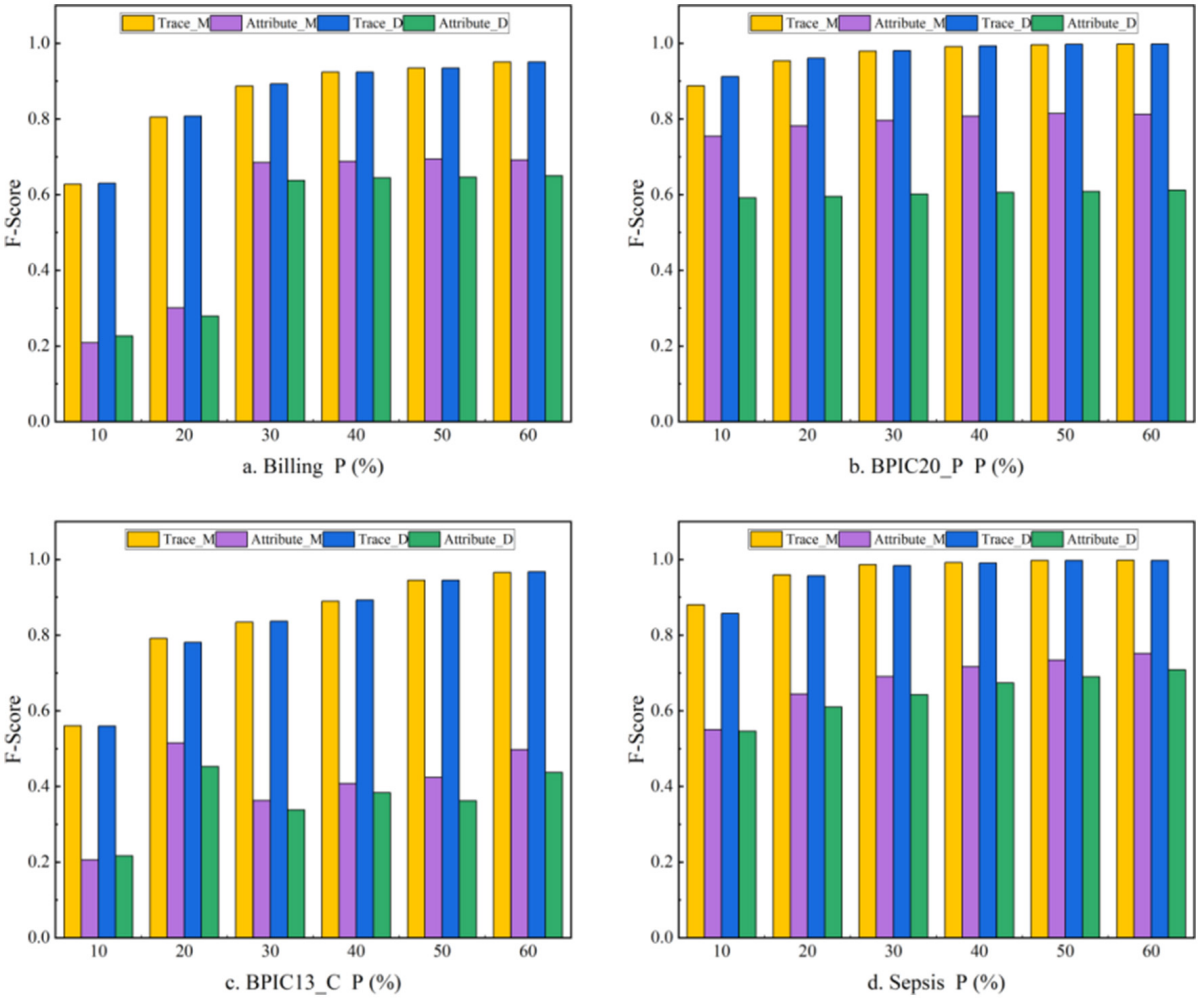
Results of ablation experiments.

The ablation experiment mainly focuses on whether to utilize behavioral constraints to construct trace graphs. MLGTAE builds a behavioral relationship matrix by extracting the footprint relationships between activities from Petri nets and then constructs local trace graphs based on the globally constructed behavioral relationship matrix. To verify the role of behavioral relationships, we eliminated this way of constructing trace graphs and adopted direct successor graphs to construct trace graphs instead. The comparison results before and after ablation are shown in Figure 10, where Trace_M and Attribute_M are the trace-level and attribute-level results obtained by MLGTAE on the four datasets, respectively, while Trace_D and Attribute_D are the results obtained by constructing trace graphs using direct successor graphs. It can be observed that the results before ablation are generally better than those after ablation on these four datasets, especially at the attribute level, where the F1-score of MLGTAE has significantly improved. This also answers question (2), indicating that the trace graph construction method guided by behavioral relationships achieves better results than the direct successor graph method.

#### 5.4.3 Hyperparameter analysis

To answer question (3), the impact of the hidden layer dimension and the number of attention heads on the anomaly detection results is discussed below. As shown in Table 5, we considered three levels of hidden layer dimensions (32, 64, 256). The results obtained on the Hotel Billing and Sepsis datasets indicate that, under different anomaly insertion ratios (10%, 20%, 30%), as the number of hidden layers increases, the anomaly detection performance improves. The hidden layer dimension has a significant impact on the anomaly detection results. However, it is worth noting that an appropriate hidden layer dimension should be selected. A larger dimension requires more parameters and significantly increases the computational load. If the dimension is too large, it may lead to overfitting.

**Table 5:** F1-scores of trace levels obtained with different hidden layer dimensions.

**Data parameter**	**Billing (10)**	**Billing (20)**	**Billing (30)**	**Sepsis (10)**	**Sepsis (20)**	**Sepsis (30)**
Hidden dim (32)	0.682	0.851	0.927	0.854	0.958	0.984
Hidden dim (64)	0.699	0.858	0.928	0.864	0.958	**0.985**
Hidden dim (256)	**0.788**	**0.885**	**0.935**	**0.880**	**0.959**	**0.985**

The bold values indicate the outstanding results in the experiment.

As shown in Table 6, the impact of the number of attention heads on the results of anomaly detection was explored. Three levels of attention heads (1, 4, 8) were considered. The results obtained on the Hotel Billing and BPIC20_P datasets indicate that under different anomaly insertion ratios (10%, 20%, 30%), the anomaly detection results did not change significantly with the variation in the number of attention heads. The maximum change was 0.013. The reason for this phenomenon is that MLGTAE adopts a multi-encoder and multi-decoder form, which enables each corresponding encoder to focus on the corresponding node features. Even if the number of attention heads is increased, the processing is still carried out on the corresponding features, so no significant changes occur.

**Table 6:** F1-scores of trace levels obtained with different numbers of attention heads.

**Data level**	**Billing (10)**	**Billing (20)**	**Billing (30)**	**BPIC20_P (10)**	**BPIC20_P (20)**	**BPIC20_P (30)**
Nhead (1)	**0.788**	0.885	**0.935**	0.887	0.953	**0.979**
Nhead (4)	0.780	0.881	0.934	0.903	**0.956**	0.978
Nhead (8)	0.775	**0.887**	**0.935**	**0.906**	0.954	0.977

The bold values indicate the outstanding results in the experiment.

## 6 Conclusion and future work

Aiming at the abnormal situations existing in the execution process of hotel operation processes, this paper proposes a multi-perspective business operation process anomaly detection method based on Graph Transformer and autoencoder (MLGTAE). MLGTAE extracts the global behavioral profile relationship from the Petri net, and then uses the obtained behavioral relationship to standardize the generation of trace graphs. The generated trace graphs and multiple case attributes are coupled to output multi-perspective trace graphs specific to the current trace. The obtained trace graphs are input into the autoencoder to achieve anomaly identification at the trace level and multiple attribute levels. The autoencoder's encoder is a Graph Transformer (combining Transformer and GCN). The Transformer allows for handling long sequences, while the GCN integrates the behavioral relationships among process activities. The synergy of GCN and Transformer provides MLGTAE with robust multi-perspective representation capabilities for both behavior and attributes. The decoder is a multi-layer LSTM, and before inputting the encoder output into the decoder, an additive attention process is required to achieve interaction among multiple encoder outputs. Finally, the anomaly detection results are obtained based on the decoder output.

The future work mainly has two aspects. The first aspect is to consider the rule constraint relationship of the attribute itself for different attributes of the same case to formulate a specific graph structure, rather than only taking the behavioral relationship between activities as the orientation to standardize the uniform structure information. The other aspect is to achieve dynamic and forward-looking anomaly prediction in the online process environment when different activities of different cases occur in real time, which is also a direction worth studying. At the same time, multi-hotel support will be further expanded, more advanced AI technologies will be introduced, and mobile applications will be optimized to improve the intelligence level and user experience of the system. The research in this paper provides a reliable anomaly handling solution for hotel operations.

## Data Availability

The original contributions presented in the study are included in the article/supplementary material, further inquiries can be directed to the corresponding author.
